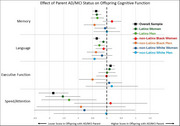# Socioeconomic, Biomarker, Genetic, and Cardiovascular Drivers of the Effect of Parental Alzheimer's Disease and MCI Status on Cognitive Function in Middle Age

**DOI:** 10.1002/alz70860_102204

**Published:** 2025-12-23

**Authors:** Jennifer J. Manly, Benjamin D. Huber, Miguel Arce Rentería, Justina F. Avila, Emily Hokett, A. Zarina Kraal, Patrick J. Lao, Jessica Mazen, Jeffrey D. Pyne, Dominika Seblova, Indira C. Turney, Edric D. Winford, Adam Brickman

**Affiliations:** ^1^ Taub Institute for Research on Alzheimer's Disease and the Aging Brain, Columbia University, New York, NY, USA; ^2^ Department of Neurology, Vagelos College of Physicians and Surgeons, Columbia University, New York, NY, USA; ^3^ Second Faculty of Medicine Charles University, Prague, Czech Republic; ^4^ National Institutes of Health / National Institute on Aging, Baltimore, MD, USA

## Abstract

**Background:**

Identification of mechanisms that link midlife cognitive function with parental history of AD among people racialized as Black, White, and Latinx may reveal potential interventions to reduce disparities in cognitive aging and dementia. Direct measurement of parental cognition is critical, because underdiagnosis of ADRD in minoritized populations may introduce bias in family studies. We evaluated the effect of parental AD/MCI history on middle age cognitive function, and determined whether genetic, biomarker, cardiovascular, or socioeconomic factors account for parental risk.

**Method:**

Parents (*n* = 1124) were participants in the Washington Heights Columbia Aging Project (WHICAP), where diagnosis (48% normal cognition, 26% AD, 26% MCI) was established via consensus conference at baseline and follow‐up waves that occur approximately every 2 years. Cognitive outcomes for their adult offspring (*n* = 1799, mean age = 56.1, SD = 11.0; range = 27 ‐ 91; 66% Latinx, 19% Black, 14% White) for executive function, language, speed/attention, and memory domains were developed using a confirmatory factor analysis and scores from an extensive neuropsychological battery. Regression analyses controlling for offspring age, sibling relatedness, and remote/in‐person assessment determined the association of parent status (AD/MCI vs normal) with offspring cognition. Subsequent analyses considered whether characteristics of parents (sex/gender, educational attainment, income, occupational prestige, APOE4 status, and cardiovascular burden) and offspring (cardiovascular burden and plasma levels of amyloid beta 42:40 ratio, pTau‐181, NfL, and GFAP) accounted for the relationship between parental cognitive impairment and offspring cognition.

**Result:**

Offspring of parents with AD/MCI had worse speed/attention, language, and memory than offspring of parents without AD/MCI. The effect of parent AD/MCI on offspring language scores was similar across race, ethnicity, and gender, but varied by group for attention, executive functioning, and memory. Accounting for parent education or income attenuated the effect of parent AD/MCI on offspring cognition by about 50%. None of the other genetic, biomarker, or cardiovascular variables in parents or offspring explained the effect of parent status on offspring cognition.

**Conclusion:**

In a community representative cohort, parent socioeconomic position was a significant driver of the effect of parent AD/MCI on poorer cognition.